# Supplementary material for: COZOID: contact zone identifier for visual analysis of protein-protein interactions
Source: BMC Bioinformatics. 2018 Apr 6;19:125. doi: 10.1186/s12859-018-2113-6 (PMC5889581; doi:10.1186/s12859-018-2113-6)
Supplement: Supplementary file 2 — Software build. Executable binary file of the software tool. (ZIP 118,784 kb) [file 12859_2018_2113_MOESM2_ESM.zip › cozoid/platform/docs/testng-6.5.1-javadoc.zip!/org/testng/IModuleFactory.html]

IModuleFactory (TestNG)


---


|  |  |  |
| --- | --- | --- |
| **Overview** \| **Package** \| **Class** \| **Tree** \| **Deprecated** \| **Index** \| **Help** | |  |
| **PREV CLASS**   **NEXT CLASS** | **FRAMES**    **NO FRAMES**     **All Classes** |  |
| SUMMARY: NESTED \| FIELD \| CONSTR \| METHOD | DETAIL: FIELD \| CONSTR \| METHOD |  |


---


## org.testng Interface IModuleFactory

---

``` public interface IModuleFactory ```

This interface is used by the moduleFactory attribute of the @Guice
annotation. It allows users to use different Guice modules based on the test
class waiting to be injected.

**Author:**
:   Cedric Beust

---

| **Method Summary** | |
| --- | --- |
| `com.google.inject.Module` | `createModule(ITestContext context, java.lang.Class<?> testClass)` |

**Method Detail**

### createModule

```
com.google.inject.Module createModule(ITestContext context,
                                      java.lang.Class<?> testClass)
```

:   **Parameters:**: `context` - The current test context: `testClass` - The test class **Returns:**: The Guice module that should be used to get an instance of this test class.


---


|  |  |  |
| --- | --- | --- |
| **Overview** \| **Package** \| **Class** \| **Tree** \| **Deprecated** \| **Index** \| **Help** | |  |
| **PREV CLASS**   **NEXT CLASS** | **FRAMES**    **NO FRAMES**     **All Classes** |  |
| SUMMARY: NESTED \| FIELD \| CONSTR \| METHOD | DETAIL: FIELD \| CONSTR \| METHOD |  |


---
